# A Scary Tale of a Twisted Storm: A Case Report of Torsades De Pointes in Acquired Long QT Syndrome

**DOI:** 10.7759/cureus.86577

**Published:** 2025-06-23

**Authors:** Andrew S Dzebu, Edem Draffor, Orlando Henriquez Italin

**Affiliations:** 1 Cardiothoracic Centre, Ho Teaching Hospital, Ho, GHA; 2 Internal Medicine, Ho Teaching Hospital, Ho, GHA; 3 Cardiology, Hermanos Ameijeiras Hospital, Havana, CUB

**Keywords:** electric storm, pacing, syncope, torsade de pointes, ventricular tachycardia

## Abstract

*Torsade de pointes* (TdP, meaning “twisting of the points”) is a polymorphic ventricular tachycardia (VT) characterized by twisting QRS complexes around the isoelectric line, typically associated with prolonged QT intervals from congenital or acquired causes. A 64-year-old woman with hypertension presented with recurrent syncope and bradycardia. A 12-lead electrocardiogram (ECG) revealed a 2:1 atrioventricular block (AVB) and a prolonged corrected QT (cQT) interval (544 ms). Holter monitoring showed repeated episodes of TdP correlating with her symptoms. This case underscores prompt recognition and treatment to prevent sudden cardiac death - in this case, by electrolyte modulation.

## Introduction

*Torsade de pointes* (TdP), first described by Dessertenne in 1966, means “twisting of the points” in French [1,2]. It is a polymorphic ventricular tachycardia (VT), characterized by a distinctive variation of QRS amplitudes around the isoelectric line, associated with long QT syndromes (LQTS).

LQTS can be acquired or congenital. Acquired causes include drugs, bradycardia, myocardial ischemia, hypothyroidism, and electrolyte disorders, among others. At least 17 genetic variants of the congenital type, often diagnosed in childhood, have been identified to date, including the Romano-Ward, Jervell & Lange-Nielsen, Andersen-Tawil, and Timothy syndromes [1,3].

We present a patient with recurrent syncope. A 12-lead electrocardiogram (ECG) noted high-grade atrioventricular block (AVB) with a ventricular rate of about 40 bpm, a persistently prolonged corrected QT (cQT) interval (544 milliseconds, as calculated by the Bazett formula), and the characteristic torsion of the points on Holter monitoring. A prolonged cQT interval increases the clinical risk of syncope and cardiac arrest.

## Case presentation

We present a 64-year-old female with a history of well-controlled hypertension, who was referred to our emergency room on account of syncope. These episodes of loss of consciousness, which started a day before admission, were preceded by lightheadedness and palpitations, as observed by the patient. The first episode occurred while she was standing, resulting in a fall without significant consequences. Witnesses report short episodes of unresponsiveness, lasting several seconds (10-15 seconds). She regains consciousness thereafter and does not have any sequelae. This recurs a few times within an hour (approximately four per hour). She has since preferred to stay in bed for fear of the traumatic consequences of a fall. She took amlodipine for hypertension but denies taking any other medication, herbal products, or illicit drugs.

During the presentation, she was conscious and oriented. Cardiovascular examination showed a blood pressure of 150/90 mmHg and a pulse rate of 42 bpm. Physical examination was otherwise unremarkable. A 12-lead ECG was significant for a 2:1 AVB. Laboratory investigations were unremarkable, including complete blood count, renal function tests, electrolytes, and thyroid function tests. Echocardiography was significant only for calcific mitro-aortic valves (without stenosis or regurgitation). This is age-related and likely associated with degenerative changes in the conduction system, which may manifest as AVB, as in her case.

**Figure 1:**
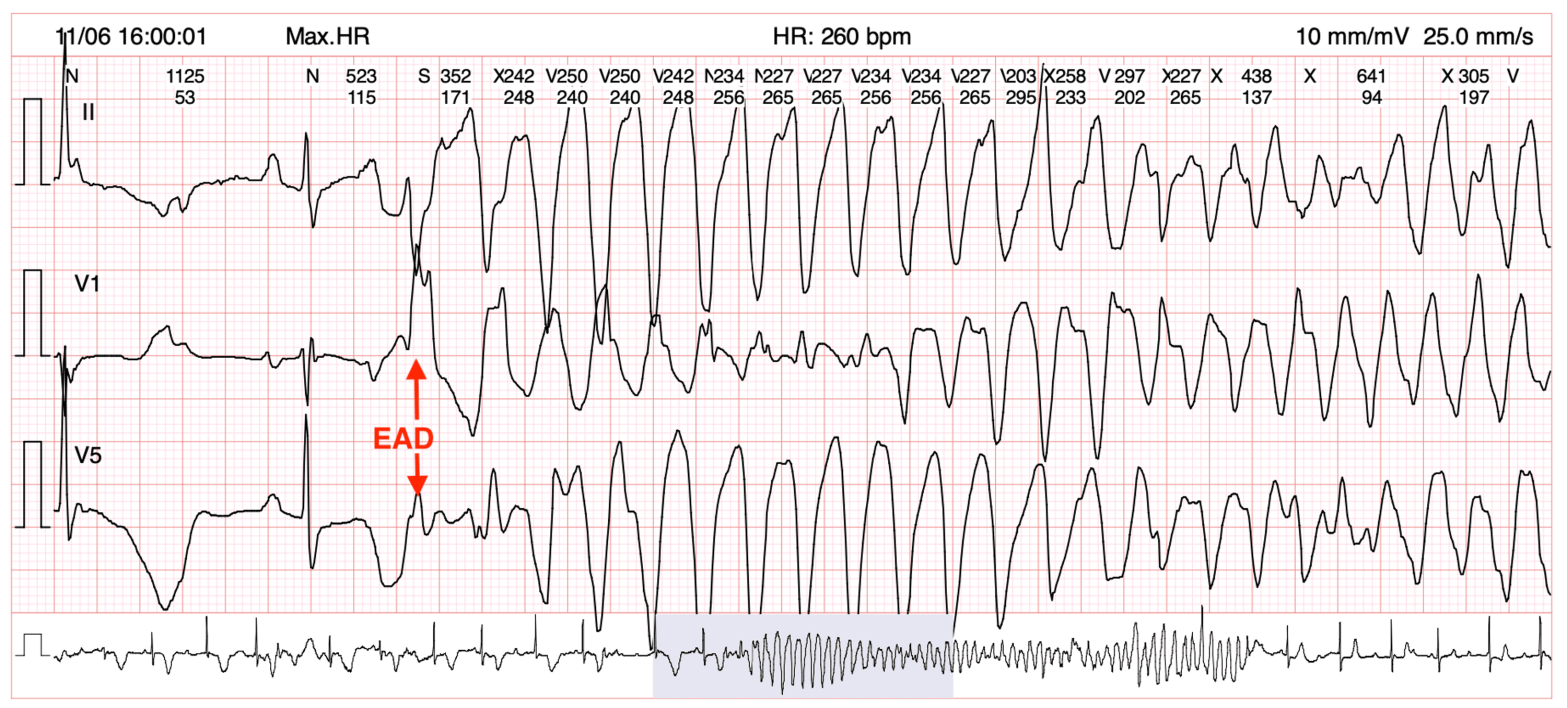
Extract from the Holter monitor Note the last normal QRS complex with a long QT interval and an EAD, which appears as an R-on-T phenomenon, followed by subsequent degeneration into the characteristic TdP. EAD, Early afterdepolarization; TdP, *Torsade de pointes*

Holter monitoring revealed persistent bradycardia, with an average heart rate of 45 bpm, and >95% of beats <55 bpm. The cQT interval peaked at 500 ms at a heart rate of 50 bpm. There were various episodes of polymorphic wide-complex tachycardia, with a maximum rate of 260 bpm, with oscillatory changes in the amplitude of the QRS complex (Figure 1). These episodes correlated with episodes of syncope. The arrhythmia self-terminated. This case meets the criteria for bradycardia-related acquired LQTS.

With this information, magnesium sulfate (MgSO_4_) was given intravenously. Isoproterenol and temporary transvenous pacing were unavailable, so transcutaneous overdrive pacing was attempted unsuccessfully due to failure to capture. Spironolactone was given at 25 mg daily for electrolyte modulation (of potassium) to improve myocardial repolarization. After about 12 hours of MgSO_4_ infusion, episodes of syncope ceased. The patient maintained a 2:1 AVB, with a normal cQT interval. MgSO_4_ was stopped. The patient remained asymptomatic (no loss of consciousness, lightheadedness, or palpitations). She could now get out of bed and do basic self-care activities. Permanent pacing was discussed with the patient, but could not be implemented due to a lack of financial access (out-of-pocket payment required). The patient was discharged and followed up closely for the past year. She’s been on spironolactone 25 mg daily, together with amlodipine 5 mg for essential hypertension. A year after hospital discharge, she still has a 2:1 AVB and can perform daily activities with minimal fatigue. We consider this a clinical paradox: this patient, with an ECG, is still showing 2:1 AVB and a normal cQT interval (450 ms) without recurrent TdP. While no reversible etiology was found, including electrolyte imbalance and persistence of a 2:1 AV block, we believe electrolyte modulation using MgSO_4_ and spironolactone was key in reverting the electrical storm presented by the patient, probably by improved repolarization and/or adaptation. This detail may be useful for further investigation in the field of cardiovascular electrophysiology. 

## Discussion

TdP is a polymorphic VT with an undulating QRS axis that usually occurs in LQTS. It is triggered by an early afterdepolarization (EAD) and sustained in a substrate with a reentry mechanism, such as inhomogeneous repolarization across layers of the ventricle. The sub-epicardium has the shortest action potential duration (APD), the mid-myocardial layer has the longest APD, and the sub-endocardial layer has an intermediate APD. This transmural gradient creates the substrate for reentry. This substrate is vulnerable to an EAD which, when it occurs, can lead to a premature ventricular contraction (PVC) - in the case of the Purkinje fibers - potentially infringing on the underlying substrate of heterogeneous repolarization to initiate a polymorphic reentrant VT. Excess transmural heterogeneity of the APD is thought to provide the unidirectional block and functional reentry circuits that perpetuate TdP [2,3].

Congenital LQTS is one cause of ventricular arrhythmia in a “structurally normal” heart. It presents clinically as syncope, lightheadedness, palpitations, or, rarely, as an electrical storm [3,4]. It is diagnosed (i) in the presence of a cQT interval >500 ms on repeated 12-lead ECGs, (ii) a cQT interval between 480 and 499 ms and syncope, or (iii) a Swartz score >3.5 points. Congenital causes are heritable and include at least 17 types, such as Romano-Ward syndrome and Jervell & Lange-Nielsen syndrome. In acquired LQTS, a prolonged cQT interval is required in a heart that may be structurally normal or abnormal, and includes causes such as drugs, coronary artery disease, and degenerative disease of the conduction system, resulting in bradycardia. Whether congenital or acquired, ion channels in cardiomyocytes malfunction in LQTS, reducing repolarization currents via potassium channels or causing excessive depolarization through late-sodium currents. This leads to late inactivation of calcium channels and, therefore, excess influx of calcium, resulting in EAD [1,3].

**Figure 2:**
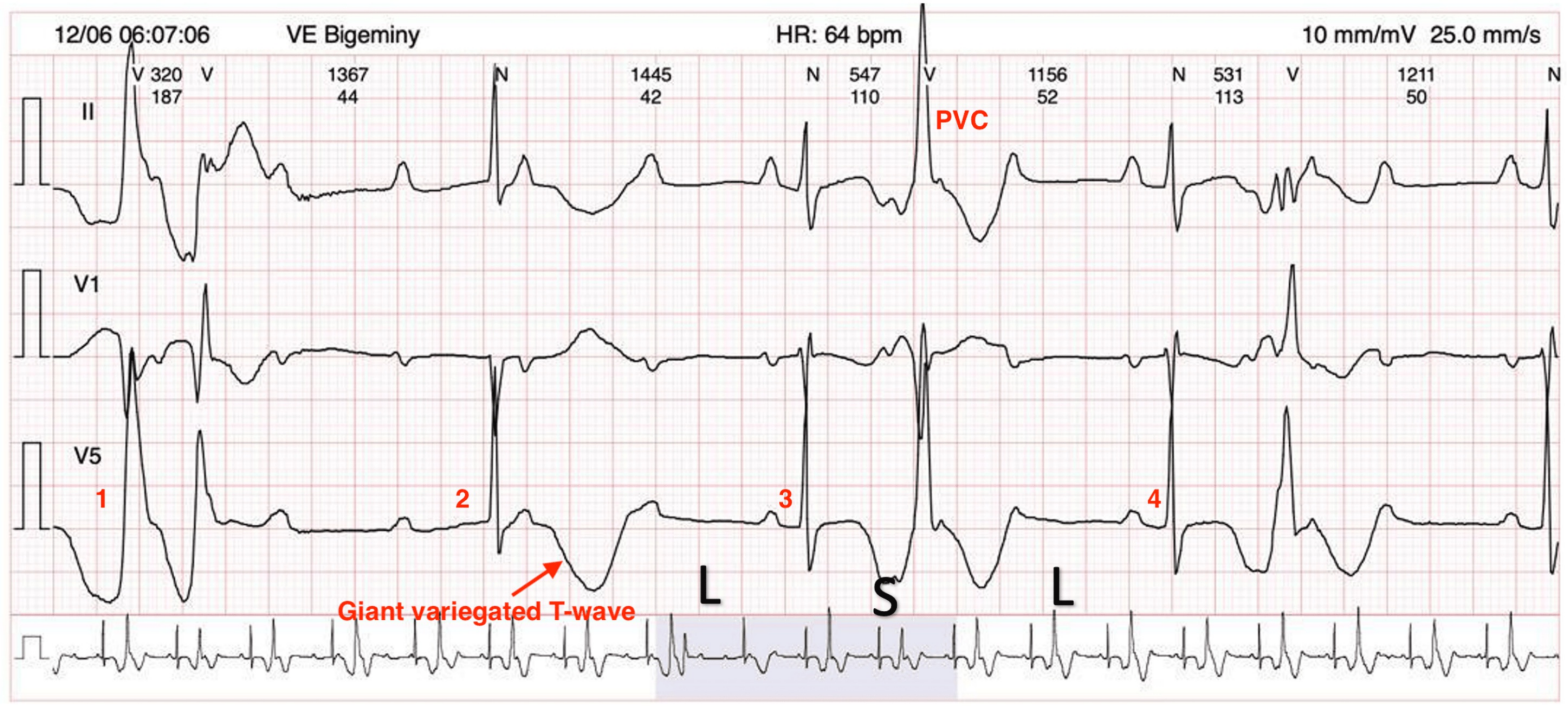
Extract from the Holter monitor Grade 3 atrioventricular block, alternating QT intervals, giant variegated T-waves, and polymorphic PVCs are evident. They appear as a couplet in beat 1, with long coupling intervals, and are bigeminal in beats 3 and 4. Long-short-long cycles are also evident. These features have been associated with sudden cardiac death. 1, Beat 1; 2, Beat 2; 3, Beat 3; 4, Beat 4; L-S-L, Long-short-long cycle; PVC, Premature ventricular contraction

The onset of TdP requires two fundamental conditions: (i) the first beat of the arrhythmia denotes an EAD that has reached trigger potential at the late phase of a prolonged action potential, and (ii) an abnormal QT interval that is unable to accommodate sudden heart rate changes - be it tachycardia-dependent (mainly in infants or LQTS-1) or pause-dependent (in LQTS-2, LQTS-3, and acquired LQTS) [1]. Acquired LQTS typically initiates TdP with a characteristic short-long-short sequence of R-R intervals (Figure 2). In this pattern, a prolonged pause intensifies QT interval prolongation, which increases the risk of TdP by enhancing transmural dispersion of repolarization. This sequence usually begins with a PVC, a compensatory pause, and another PVC that falls on the T wave. Although this R-on-T PVC can trigger TdP, its coupling interval is not as short as in idiopathic ventricular fibrillation, due to the prolonged QT interval. Additionally, TdP in acquired LQTS is often linked to bradycardia or frequent pauses - a phenomenon called “pause-dependent LQTS” [3,5,6].

In patients with acquired LQTS, such as this patient in question, there is a maladaptation of the QT interval to the deceleration of the heart rate due to the post-extrasystolic pause. The post-extrasystolic pause is represented by a giant, bizarre T-wave.

Acquired reversible etiologies may include electrolyte imbalance; drugs (see https://www.crediblemeds.org/); myocardial disease (e.g., myocardial ischemia and takotsubo cardiomyopathy); autonomic influences; hypothermia; hypothyroidism; pheochromocytoma; and intracranial bleeding. Acquired irreversible causes include bradycardia complicating AVB (degenerative or iatrogenic) [3,5-7].

Treatment of TdP must be comprehensive: (i) tackling the etiology, such as withdrawing offending drugs that prolong the QT interval; (ii) correcting electrolyte imbalance, if any (such as hypokalemia, hypomagnesemia, and hypocalcemia), with magnesium (serum level of 2-3 mmol/L) and potassium (serum level of 4.5-5 mmol/L) supplementation. MgSO_4_ suppresses TdP, presumably by attenuating EADs that could trigger TdP. This EAD suppression ability has been attributed to its calcium channel-blocking effects but may also be mediated by a reduction in the late component of the sodium current; (iii) increasing heart rate using pharmacological agents (e.g., isoproterenol) or overdrive pacing in pause-dependent LQTS. Permanent pacing is required where no reversible etiology is present, such as in symptomatic AVB. An implantable cardioverter-defibrillator may be necessary, especially in congenital LQTS; and (iv) beta-blocker therapy, to stabilize the heterogeneous transmural gradient, is indicated in congenital LQTS [1,3,5,6]. Promising pharmacological therapy includes late sodium channel inhibitors (e.g., eleclazine and ranolazine), potassium channel openers (e.g., nicorandil and retigabine), calcium channel blockers (e.g., verapamil), and gene therapy [8]. 

## Conclusions

TdP is a polymorphic VT characterized by twisting QRS complexes, typically associated with prolonged QT intervals from congenital or acquired causes. A 64-year-old woman with hypertension presented with recurrent syncope and bradycardia. ECG revealed a 2:1 AVB and a prolonged cQT interval (544 ms). Holter monitoring showed repeated episodes of TdP correlating with her symptoms. No reversible causes were found. MgSO₄ infusion resolved the arrhythmia, though transcutaneous overdrive pacing failed. She was stabilized on spironolactone (for potassium modulation and improved myocardial repolarization), and permanent pacing was considered but not pursued due to cost. She remained in advanced (2:1) AVB, maintaining a risk of progression to complete AVB, syncope, or cardiac death. However, she has been able to perform activities of daily living one year after hospital discharge. Electrolyte modulation may have resolved the ventricular arrhythmia in this patient.

TdP is triggered by EAD and sustained by transmural repolarization gradients. Acquired causes include bradycardia, electrolyte imbalances, and certain drugs. Diagnosis relies on a prolonged cQT interval and clinical history. Management involves addressing underlying causes, correcting electrolytes, increasing heart rate pharmacologically or via pacing, and considering defibrillators in high-risk cases. Novel therapies are under development for LQTS. This case underscores the importance of prompt recognition and treatment to prevent sudden cardiac death. Scarce resources for optimal cardiovascular care in our region are also of note.
